# 

**Published:** 1894-02

**Authors:** 


CONTENTS—FEBRUARY, 1894.—VOL. IX., NO. 8.

ORIGINAL COMMUNICATIONS—

A Case of Drowning. By T. C. Osborn, M. D., Cleburne, Tex . . . 373

Some of the Hardships of the General Practitioner of Medicine, and a Suggestion for Relief. By R. P. Talley, M. D., Temple, Tex . . 378

Specific Remedies in Infectious Diseases; Translated from the German 385

ABSTRACTS OF CURRENT MEDICAL LITERATURE—

Department of Practice of Medicine. Edited by Prof. A. J. Smith, M. D., Galveston . . . . . . . . . . . . . . . . . . . . 388

Department of Therapeutics. Edited by Prof. D. Cerna, M. D., Galveston . . . . . . . . . . . . . . . . . . . . . . . . . 394

Department of Surgery. Edited by Prof. J. E. Thompson, M. D., Galveston . . . . . . . . . . . . . . . . . . . . . . . . . 398

SOCIETY NOTES—

Terrell Medical Society, 401; Eleventh International Medical Congress, 402; Pathological Section of Medico-Legal Society, 403 . . 401-3

EDITORIAL—

Conservative Gynecology, 405; An Unworthy Suspicion, 410; Ex-Confederates all Right, 411; A Travesty on Religion and Law, 412 405-12

MEDICAL NEWS AND MISCELLANY—

Interesting Items Too Numerous to Shake a Stick At . . . . . . . 413

BOOK NOTICES—

Modern Gynecology; Bushong, 420; The Theory and Practice of Medicine, etc., Whittaker, 422; A Treatise on the Principles of Pharmacy, etc., in Preparation by Kennedy and Morris, 422; Naphey's Modern Therapeutics, Smith, 422; Diseases of the Rectum and Anus, Kelsey, 425 . . . . . . . . . . . . . . . . . . . . . . . . . . 422-5

PUBLIStHERS' NOTES . . . . . . . . . . . . . . . . . . . . . . 426

